# The Effect and Optimal Parameters of Repetitive Transcranial Magnetic Stimulation on Poststroke Dysphagia: A Meta-Analysis of Randomized Controlled Trials

**DOI:** 10.3389/fnins.2022.845737

**Published:** 2022-04-28

**Authors:** Jia Qiao, Qiu-ping Ye, Zhi-min Wu, Yong Dai, Zu-lin Dou

**Affiliations:** ^1^Department of Rehabilitation Medicine, The Third Affiliated Hospital of Sun Yat-sen University, Guangzhou, China; ^2^Department of Neurosurgery, The Third Affiliated Hospital of Sun Yat-sen University, Guangzhou, China; ^3^Clinical Medical of Acupuncture Moxibustion and Rehabilitation, Guangzhou University of Chinese Medicine, Guangzhou, China

**Keywords:** poststroke dysphagia (PSD), repetitive transcranial magnetic stimulation (rTMS), randomized controlled trial, meta-analysis, parameters

## Abstract

**Objective:**

The objectives of the study were to evaluate the efficacy of repetitive transcranial magnetic stimulation (rTMS) treatment for poststroke dysphagia (PSD) and explore the optimal stimulation parameters.

**Method:**

The databases of Medline, Embase, Web of Science, and Cochrane Library were searched from the establishment to June 2021. All randomized controlled trials about rTMS treatment for PSD were enrolled. Dysphagia Grade (DG) and Penetration Aspiration Scale (PAS) were applied as the major dysphagia severity rating scales to evaluate the outcomes.

**Results:**

A total of 12 clinical randomized controlled studies were included in our study. The summary effect size indicated that rTMS had a positive effect on PSD (SMD = −0.67, *p* < 0.001). The subgroup analysis for treatment duration and different stroke stages showed significant differences (treatment duration >5 days: SMD = −0.80, *p* < 0.001; subacute phase after stroke: SMD = −0.60, *p* < 0.001). Furthermore, no significant differences were observed among the other stimulation parameter subgroups (including stimulation frequency, location, and a single stimulation time) (*p* > 0.05).

**Conclusion:**

rTMS is beneficial to the recovery of PSD patients, while an intervention of more than 5 days and in the subacute phase after stroke might bring new strategies and rational therapeutics to the treatment of PSD.

**Systematic Review Registration:**

http://www.crd.york.ac.uk/PROSPERO/, identifier: CRD42022299469.

## Introduction

Poststroke dysphagia (PSD) is a common after stroke, with an incidence ranging from 27 to 78% (Rofes et al., 2013; Benjamin et al., 2018; Zhong et al., 2021). The clinical complications of PSD include malnutrition, aspiration pneumonia, dehydration, social difficulties, etc. (Ekberg et al., 2002; Clavé et al., 2012; Kalra et al., 2015), which could lead to an increase in medical expenses and mortality, and impairment of the quality of life (Altman et al., 2010; Sasegbon and Hamdy, 2017). The regular treatment of PSD mainly includes oropharyngeal exercises, expiratory muscle training, compensation strategies (Langmore and Miller, 1994; Rogus-Pulia and Robins, 2013; Zhong et al., 2021), neuromuscular electrical stimulation (MMES) (Speyer et al., 2010; Mao et al., 2021; Zhong et al., 2021), invasive procedures, and noninvasive brain stimulation (NIBS) (Ludlow et al., 2007).

Two major NIBS techniques are repetitive transcranial magnetic stimulation (rTMS) and transcranial direct current stimulation (tDCS), which offer the advantage of relatively wide availability, comfort, convenience, and lower side effects for the PSD patient as they are noninvasive interventions. Since rTMS and tDCS can also induce long-lasting effects, these techniques can be also used as adjuvant strategies for the treatment of psychiatric disorders (such as depression and bipolar disorder) and the rehabilitation of other neurological deficits (Fregni and Pascual-Leone, 2007; Sandrini et al., 2015; De Risio et al., 2020; Konstantinou et al., 2021; Pateraki et al., 2022).

The rTMS is an electromagnetic technique in which rapidly changing magnetic fields are used to induce the change in electrical currents and synaptic plasticity in the brain continuously (Pascual-Leone et al., 1998; Ridding and Rothwell, 2007). These changes can further alter cortical excitability and associated behaviors (Fiske et al., 2009; Schneider et al., 2010). Over the past decade, rTMS as a practical technique has proved effective and been widely used to promote the recovery of PSD (Verin and Leroi, 2009). The balance of neural activities between the bilateral cerebral hemispheres is dramatically disturbed and impaired after stroke—which is caused by the altered corpus callosum suppression, while rTMS could be used to restore this balance and improve the swallowing function (Gow et al., 2004; Du et al., 2016; Ünlüer et al., 2019; Zhong et al., 2021).

Various stimulation parameters in rTMS were demonstrated to be highly related to different effects, including intensity, frequency, threshold, etc. (Khedr et al., 2009; Park, 2013; Lee et al., 2015; Du et al., 2016). However, it seems that no consensus has been reached on the optimal parameters of rTMS on PSD at present (Khedr et al., 2009; Verin and Leroi, 2009). Therefore, we conducted this systematic analysis of RCT studies to assess the clinical efficacy of rTMS on PSD and explore the optimal parameters of rTMS to provide evidence for the clinical treatment of PSD.

## Materials and Methods

### Search Strategies

Medline, Embase, Web of Science, and the Cochrane Library were systematically searched from the establishment to June 2021 in English. We manually searched the related systematic reviews and further references, and searched databases with the MeSH term “dysphagia” or “swallowing disorders” or “deglutition” and “transcranial magnetic stimulation” or “rTMS” of the searching strategies for four electronic database searches. The search was limited to “randomized controlled trials,” and the full search strategy is detailed in the Supplementary Appendix. The retrieval was based on the subject terms, keywords, or titles. References from studies with potential relevance and review articles were manually checked. This study was registered with PROSPERO (CRD42022299469).

### Inclusion and Exclusion Criteria

Studies that were included in this meta-analysis met the following criteria: (1) randomized controlled trials (RCT), including cross-over and cluster RCTs; (2) patients with PSD (confirmed by VFSS, FEES, swallowing questionnaire, or dysphagic outcome and severity scale); (3) patients enrolled in studies with age ≥18 years; (4) the treatment of the experimental group was rTMS, and the researchers provided original data or sufficient information about dysphagia that occurred pre- and posttreatment in experimental trials and control trials. Exclusion criteria were: (1) publications that did not offer original data, such as reviews, meta-analysis, systematic review; (2) publications that did not meet the inclusion criteria.

### Outcome Measures

The primary efficacy outcome was the change in dysphagia severity rating scales of the PSD from baseline to postintervention, which was measured by Dysphagia Grade (DG), Penetration Aspiration Scale (PAS) at the present study.

### Data Extraction and Quality Assessment

Data were independently extracted by two well-trained evaluators (JQ and ZLD) to review original texts, supplementary appendices, and protocols. The disagreements were solved by the third author's assistance. The study characteristics included publication year, first author, journal, and patient characteristics included diagnostic criteria, age, sample size, gender, etiology, lesion side, Barthel index, and different stroke stages. The treatment duration, stimulation frequency, location, threshold and a single stimulation time were also extracted. Furthermore, the adverse events of rTMS were included. The methodological quality of the researches was assessed in accordance with the risk of bias tool described in the Cochrane Handbook for systematic reviews of interventions (Cochrane Handbook 5.2) (Higgins and Green, 2019). Random sequence generation, allocation concealment, blinding of participants and personnel, blinding of outcome assessors, incomplete outcome data, selective reporting, and other potential risks of bias were assessed. Trials that exceeded one risk assessment were considered to have a high risk of bias. Trials with all low-risk assessments were judged to have a low risk of bias; otherwise, they were considered to be at unclear risk of bias.

### Statistical Analysis

The principal emphasis of this meta-analysis was to analyze the efficacy of rTMS for PSD and assess the optimal parameters for rTMS on PSD. The measurements were expressed as standardized mean difference (SMD) with 95% confidence intervals (CI) for continuous outcomes. The funnel plots were used to detect possible publication bias. A heterogeneity test was performed using the *I*^2^ statistic; when *I*^2^ ≥ 50%, the random-effect models were used for data analysis. Otherwise, when *I*^2^ < 50%, the fixed effect model was performed (Yang et al., 2015). *I*^2^ < 25% was recognized as low heterogeneity, 25% < *I*^2^ < 75% as moderate heterogeneity, and *I*^2^ > 75% as high heterogeneity (Ampt et al., 2018). Subgroup analyses were performed to explore the optimal parameters of rTMS on PSD, including the treatment duration, different stroke stages, stimulation frequency, a single stimulation time, stimulation location, and stimulation pattern. We also performed sensitivity analyses for efficacy outcomes, in which low-quality studies would be excluded when *I*^2^ > 50%. Analyses were conducted using Review Manager (RevMan) version 5.3 (Cochrane Collaboration, London, UK). A *p*-value <0.05 was considered statistically significant.

## Results

### Characteristics of Studies

**Figure 1:**
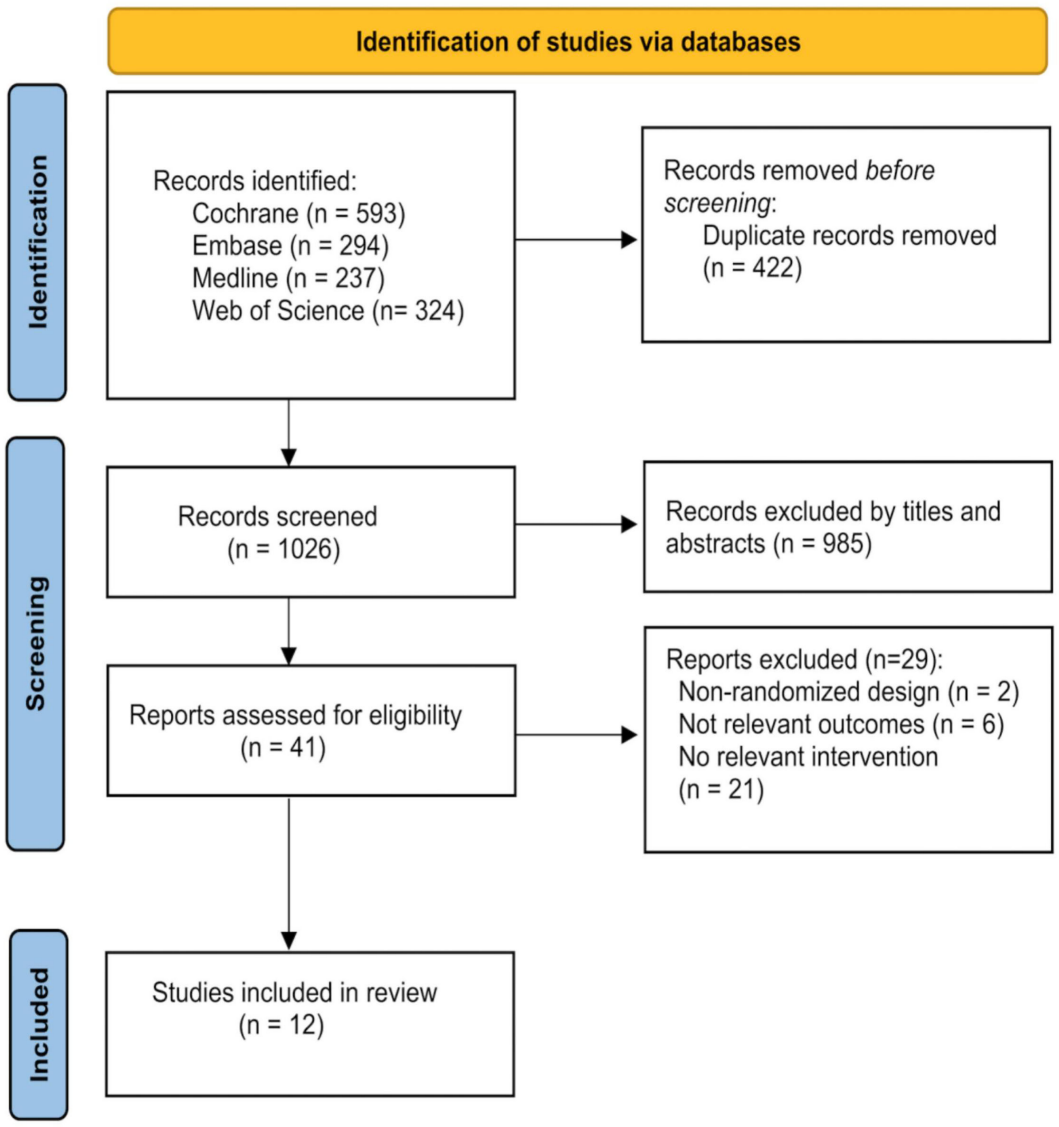
Summary of trial identification and selection.

The initial search identified 1,448 studies from four databases, of which 41 full-text articles were carefully screened by the authors. The PRISMA statement flowchart shows the process of literature screening, study selection, and reasons with exclusion (Figure 1). Only 12 high-quality articles included in our meta-analysis met the stringent criteria. The final study group was comprised of 433 patients, including 273 in the treatment group and 160 in the control group. The duration of treatment ranged from 1 to 10 days, and the stimulation frequency ranged from 1 to 10 Hz. The time for stroke onset of the included subjects ranged from 8 to 756 days. The single stimulation time varied from 5 to 20 min (Table 1).

Table 1Characteristics of included trials.

**Table:** 

**References**	**No.of patient**	**The intervention program**	**Definition of PDS**	**Different stroke stages mean ±SD day**	**Age mean ±SD year**	**Gender (M/F)**	**Etiology (H/I/Both)**	**Lesion side (R/L/Both)**
Khedr et al. (2009)	*n* = 26	rTMS (*n* = 14); T-rTMS (*n* = 12)	Dysphagic Severity scale	NA	58.9 ± 11.7; 56.2 ± 13.4	NA	NA	NA
Khedr and Abo-Elfetoh (2010)	*n* = 22	rTMS (*n* = 11); T-rTMS (*n* = 11)	Swallowing questionnaire	40.6 ± 19.6; 24.5 ± 5.6	56.1 ± 12.9; 59.3 ± 14.3	NA	NA	NA
Kim et al. (2011)x	*n* = 30	rTMSx (*n* = 10); rTMSy (*n* = 10); T-rTMS (*n* = 10)	VFSS	29.0 ± 9.9; 34.8 ± 29.7; 25.8 ± 11.2	68.2 ± 12.6; 69.8 ± 8.0; 66.4 ± 12.3	4/6; 5/5; 4/6	5/4; 4/5; 4/6	5/5; 6/4; 6/4
Kim et al. (2011)y								
Park (2013)	*n* = 18	rTMS (*n* = 9); T-rTMS (*n* = 9)	VFSS	59.9 ± 16.3; 63.9 ± 26.8	73.7 ± 3.8; 68.9 ± 9.3	5/4; 4/5	2/7; 1/8	6/3; 5/4
Lim et al. (2014)	*n* = 29	rTMS (*n* = 15); T-rTMS (*n* = 14)	VFSS	34.4 ± 10.1; 37.3 ± 16.1	62.5 ± 8.2; 66.3 ± 15.4	6/9; 6/12	5/10; 10/8	8/7; 11/7
Du et al. (2016)x	*n* = 40	rTMSx (*n* = 15); rTMSy (*n* = 13); T-rTMS (*n* = 12)	Swallowing questionnaire	8 ± 8.8; 6 ± 28.4; 9 ± 23.3	58.2 ± 2.78; 57.92 ± 2.47; 58.83 ± 3.35	13/2; 7/6; 6/6	NA	NA
Du et al. (2016)y								
Park et al. (2017)x	*n* = 33	rTMSx (*n* = 11); rTMSy (*n* = 11); T-rTMS (*n* = 11)	VFSS	28.7 ± 16.8; 29.4 ± 11.9; 46.2 ± 54.6	60.2 ± 13.8; 67.5 ± 13.4; 69.6 ± 8.6	3/8; 3/8; 4/7	4/7; 2/9; 4/7	8/3; 5/6; 5/6
Park et al. (2017)y								
Lin et al. (2018)	*n* = 28	rTMS (*n* = 13); T-rTMS (*n* = 15)	Swallowing scale;VFSS	756 ± 42.2; 648 ± 19.5	68.5 ± 12.8; 62.9 ± 12.2	1/12; 6/9	1/11/1; 4/10/1	2/7/4; 1/8/6
Tarameshlu et al. (2019)	*n* = 12	rTMS (*n* = 6); T-rTMS (*n* = 6)	MASA	22.2 ± 12; 37.3 ± 23.7	66 ± 5.55; 74.67 ± 5.92	NA	NA	NA
Ünlüer et al. (2019)	*n* = 28	rTMS (*n* = 15); T-rTMS (*n* = 13)	NA	105.93 ± 49.02; 101.38 ± 42.06	67.80 ± 11.88; 69.31 ± 12.89	6/9; 6/7	1/14; 1/12	8/7; 6/7
Cabib et al. (2020)	*n* = 24	rTMS (*n* = 12); T-rTMS (*n* = 12)	V-VST;VFSS	493.1 ± 672.4 (total)	70.0 ± 8.6	3; 9	0; 12	6; 6
Zhong et al. (2021)x	*n* = 143	rTMSx (*n* = 38); rTMSy (*n* = 36); rTMSz (*n* = 34); T-rTMS (*n* = 35)	VFSS	30 ± 70.8; 18 ± 70.4; 20 ± 23.4; 25 ± 22.6	64.47 ± 13.95; 64.67 ± 10.87; 63.18 ± 9.92; 62.34 ± 11.54	10/28; 8/28; 14/20; 17/18	18/20; 12/24; 10/24; 14/21	10/20/8; 10/14/12; 6/12/16; 5/15/15
Zhong et al. (2021)y								
Zhong et al. (2021)z

**Table:** 

**References**	**Barthel index**	**Outcome indicators**	**Treatment duration (day)**	**Frequency** **(Hz)**	**Single stimulation time**	**Stimulation location**	**Stimulation threshold**	**Remission rate**	**Adverse events (** ***n*** **)**
Khedr et al. (2009)	30 ± 25.3; 19.6 ± 21.3	DG	5	3	10 min	Affected hemisphere	100%	0/14; 0/12	NA
Khedr and Abo-Elfetoh (2010)	45.4 ± 16.0; 32.2 ± 12.2	DG	5	3	10 min	Bilateral hemisphere	130%	0/11; 0/11	NA
Kim et al. (2011)x	NA	PAS	10	5 (x)	20 min	Affected hemisphere (x)	100% (x)	0/10; 0/10;/10	None
Kim et al. (2011)y				1 (y)		Unaffected hemisphere (y)	100% (y)		
Park (2013)	34.9 ± 28.4; 34.3 ± 20.1	PAS	10	5	10 min	Unaffected hemisphere	90%	0/9; 0/9	None
Lim et al. (2014)	NA	PAS	10	1	20 min	Unaffected hemisphere	100%	5/20; 6/20	Mild headache (1)
Du et al. (2016)x	67.33 ± 22.9;	DG	5	3 (x)	10s each,40 trains in total	Affected hemisphere (x)	100% (x)	2/15; 0/13; 0/12	Transient headache (3) Tingling sensation (1)
Du et al. (2016)y	60.38 ± 17.85; 49.58 ± 25.0			1 (y)	30s each,40 trains in total	Unaffected hemisphere (y)	100% (y)		
Park et al. (2017)x	NA	PAS	10	10	10 min	Affected hemisphere (x)	90%	1/12; 0/11; 1/12	None
Park et al. (2017)y						Unaffected hemisphere (y)			
Lin et al. (2018)	NA	PAS	10	5	10 min	Left mastoid to the vagus nerve proximal	42%	0/13; 0/15	None
Tarameshlu et al. (2019)	50 ± 31.62; 24.17 ± 4.91	DG	5	1	20 min	Unaffected hemisphere	20%	0/6; 0/6	NA
Ünlüer et al. (2019)	54.53 ± 30.69; 58.08 ± 28.77	PAS	5	1	20 min	Unaffected hemisphere	90%	0/15; 2/15	Dizziness (1) Nose bleeding (2)
Cabib et al. (2020)	79.6 ± 19.9	PAS	5	5	5 min	Affected hemisphere	90%	2/14; 0/12	Syncope (1)
Zhong et al. (2021)x				5 (x)		Unaffected hemisphere (x)	110% (x)	1/39; 2/38	Transient headache (3)
Zhong et al. (2021)y	NA	PAS	10	5 (y)	20 min	Affected hemisphere (y)	110% (y)	1/35; 0/35	
Zhong et al. (2021)z				5 (z)		Cerebellum (z)	110% (z)		

*PSD, poststroke dysphagia; rTMS, repetitive transcranial magnetic stimulation; T-rTMS, the control group (none repetitive transcranial magnetic stimulation); VFSS- videofluoroscopic swallowing study; MASA, Mann Assessment of Swallowing Ability; M, Male; F, Female; H, Hemorrhage; I, Infarction; R, Right; L, Left; DG, dysphagia grade; PAS, Penetration Aspiration Scale; NA, Not mentioned in the original article; V-VST, volume-viscosity swallowing test; x,y,z, the different group; Mean, Average volume reduction of upper extremities; SD, Sample standard deviation*.

### Adverse Events

A total of five studies reported the presence of discomfort after rTMS, including mild or transient headache, tingling sensation, dizziness, nose bleeding, and syncope (Lim et al., 2014; Du et al., 2016; Ünlüer et al., 2019; Cabib et al., 2020; Zhong et al., 2021). To sum up, 7 of 273 participants (2.5%) had mild or transient headache, 1 (0.4%) had tingling sensation, 1 (0.4%) had dizziness, 2 (0.7%) had nose bleeding, and 1 (0.4%) with syncope after rTMS intervention. Reassuringly, four studies reported no adverse events (Kim et al., 2011; Park, 2013; Park et al., 2017; Lin et al., 2018), whereas the remaining studies did not mention the adverse events after rTMS (Khedr et al., 2009; Khedr and Abo-Elfetoh, 2010; Tarameshlu et al., 2019).

### Efficacy Outcomes

**Figure 2:**
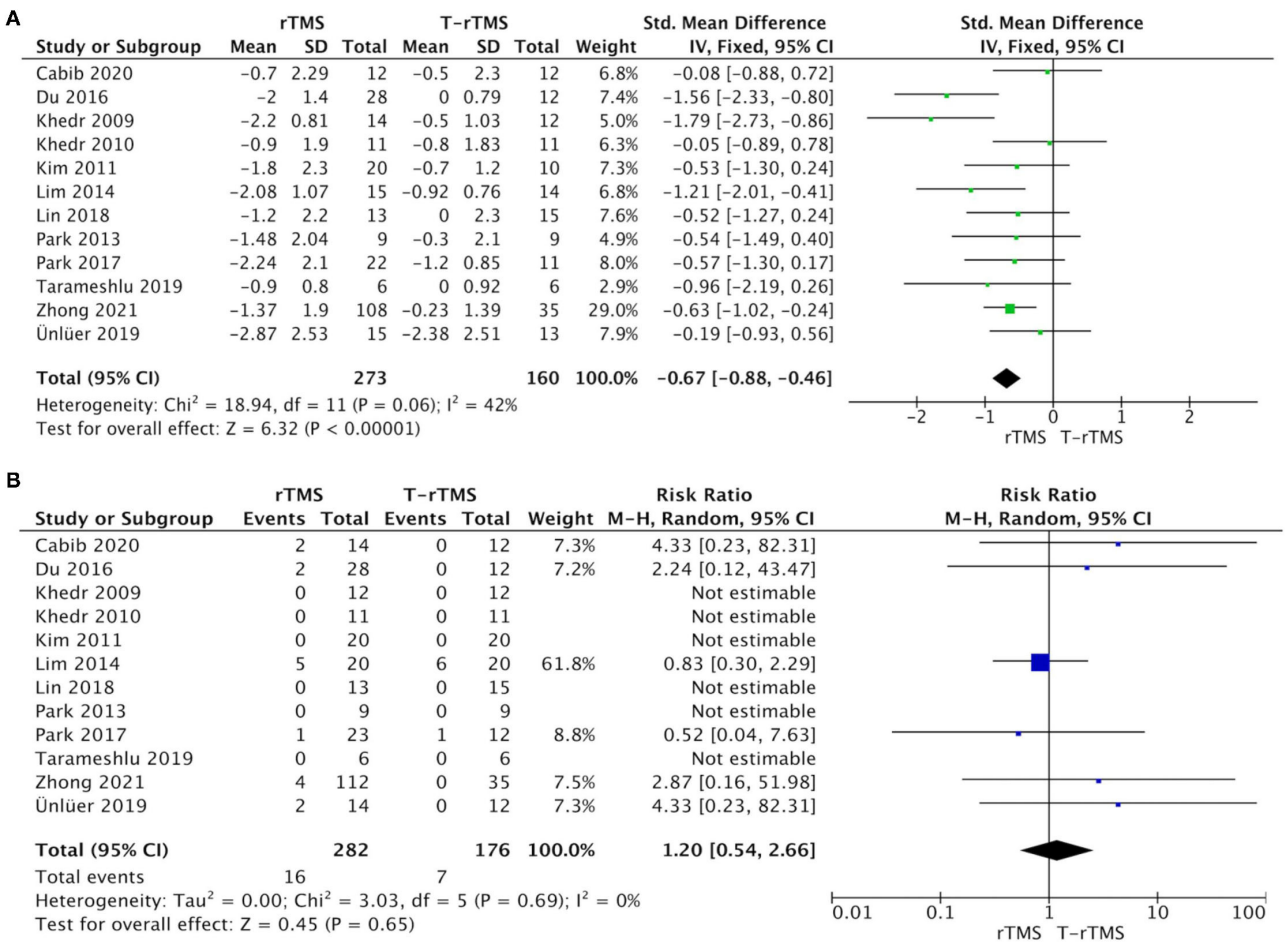
Primary efficacy outcomes and acceptability outcomes. **(A)** Forest plot of the standardized mean difference (SMD) for change scores in dysphagia severity rating scales. **(B)** Forest plot of risk ratios (with 95% confidence intervals) of dropout for any reason.

The overall pooled SMD analyzed by fixed model showed a significant advantage of rTMS interventions compared with control conditions (SMD = −0.67; 95% CI −0.88 to −0.45, *p* < 0.001), with moderate heterogeneity (*I*^2^ = 42%, *p* = 0.06), suggesting that the changes in PAS and DG in the rTMS group were much better than those in the control conditions (Figure 2A). Although outcome analysis indicated that 16 participants dropped out in the rTMS group compared with seven participants in the control conditions (Figure 2B), there was no statistical difference between the two groups (RR = 1.20, 95% CI 0.54–2.66, *p* = 0.65), with low heterogeneity (*I*^2^ = 0%, *p* = 0.69).

### Subgroup Analyses

We conducted multiple subgroups to explore the optimal parameters for rTMS on PSD, including the treatment duration (>5 vs. <5 days), different stroke stages (subacute vs. recovery phase), stimulation frequency (1 vs. ≥3 Hz), single stimulation time (≤10 vs. >10 min), stimulation location (affected vs. unaffected hemisphere), stimulation pattern (high frequency in the affected hemisphere, high frequency in the unaffected hemisphere, low frequency in the unaffected hemisphere), and age (≤65 vs. >65 years).

#### Treatment Duration

**Figure 3:**
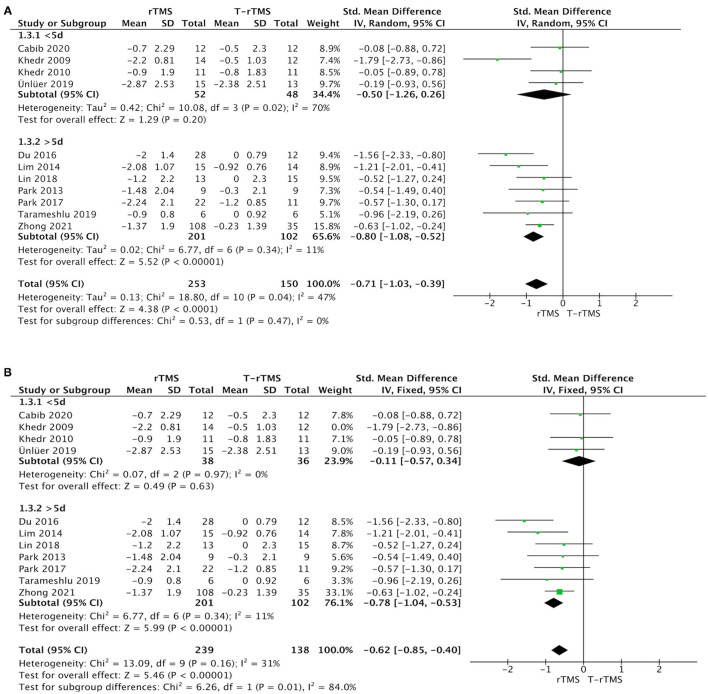
Subgroup analysis of primary outcomes. **(A)** Forest plot of the standardized mean difference (SMD) for change scores in dysphagia severity rating scales by the treatment duration. **(B)** Sensitive analysis (excluded the Khedr et al., 2009). PSD, poststroke dysphagia; rTMS, repetitive transcranial magnetic stimulation; T-rTMS, the control group; Mean, Average volume reduction of upper extremities; SD, Sample standard deviation.

Duration subgroup analysis demonstrated that treatment >5 days showed a higher effect size (SMD = −0.80; 95% CI, −1.08 to −0.52, *p* < 0.001), with low heterogeneity (*I*^2^ = 11%, *p* = 0.34) than the control conditions. However, the result in the <5-day group was opposite (SMD = −0.50; 95% CI, −1.26 to 0.26, *p* = 0.20), showing a moderate heterogeneity (*I*^2^ = 70%, *p* = 0.02) (Figure 3A).

#### Different Stroke Stages

**Figure 4:**
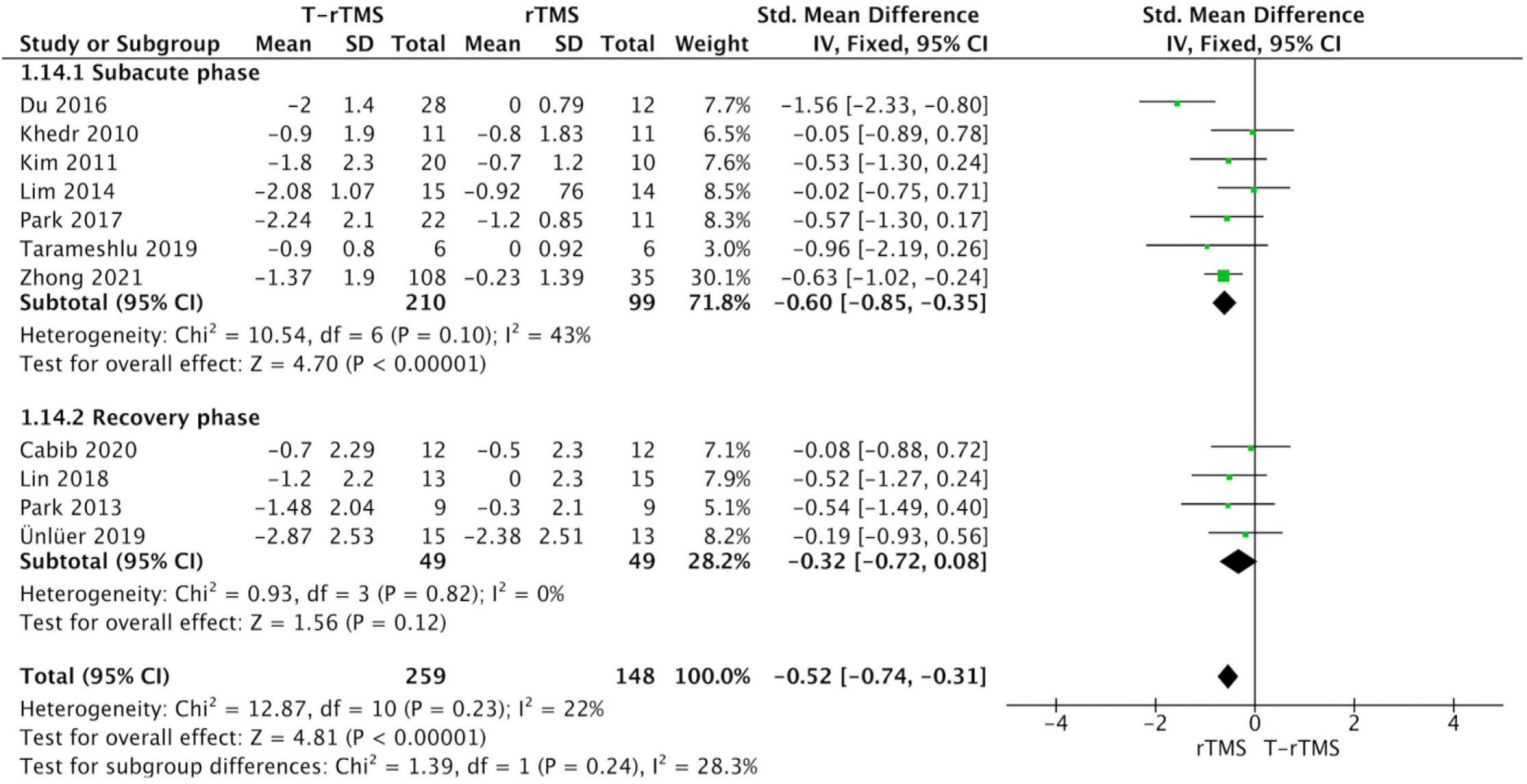
Subgroup analysis of primary outcomes: forest plot of the standardized mean difference (SMD) for change scores in dysphagia severity rating scales by the different stroke stages. PSD, poststroke dysphagia; rTMS, repetitive transcranial magnetic stimulation; T-rTMS, the control group; Mean, Average volume reduction of upper extremities; SD, Sample standard deviation.

Stroke stage subgroup analysis showed that in the subacute phase (<60 days) after stroke (Bath et al., 2018), rTMS group was significantly more beneficial than control conditions (SMD = −0.60; 95% CI, −0.85 to −0.35, *p* < 0.001), with moderate heterogeneity (*I*^2^ = 43%, *p* = 0.10). However, rTMS at the recovery phase (>60 days) failed to show a higher effect size than the control conditions (SMD = −0.32; 95% CI, −0.72 to 0.08, *p* = 0.12), with low heterogeneity (*I*^2^ = 0%, *p* = 0.82) (Figure 4).

#### Single Stimulation Time

No significant difference was found between ≤10- and >10-min groups (*p* = 0.77). According to the single stimulation time, studies in which there were ≤10 min (SMD = −0.71; 95% CI, −1.02 to −0.40, *p* < 0.001) and studies with >10 min (SMD = −0.64; 95% CI, −0.93 to −0.36, *p* < 0.001) both showed a higher effect size than the control conditions (Table 2).

**Table 2:** Meta-analyses of studies examining the effects of rTMS interventions on mean age, single stimulation period, location and pattern compared with control conditions: overall results and subgroup analyses.

**Characteristics**	**Number of contrast groups**	**SMD**	**95%CI**	***I* ^2^**	***P* value**	**Subgroup difference(*p*)**
**Mean age**						
≤65 years	6	−0.93	−1.40, −0.46	62%	*p* = 0.001	*p* = 0.08
>65 years	6	−0.41	−0.75, −0.40	0%	*p* = 0.02	
**Single stimulation time**						
≤10 min	7	−0.71	−1.02, −0.40	60%	*p* < 0.00001	*p* = 0.77
>10 min	5	−0.64	−0.93, −0.36	0%	*p* < 0.00001	
**Stimulation location**						
Affected hemisphere	6	−0.73	−1.21, −0.26	46%	*p* = 0.002	*p* = 0.28
Unaffected hemisphere	6	−1.07	−1.45, −0.69	0%	*p* < 0.00001	

#### Stimulation Frequency

**Figure 5:**
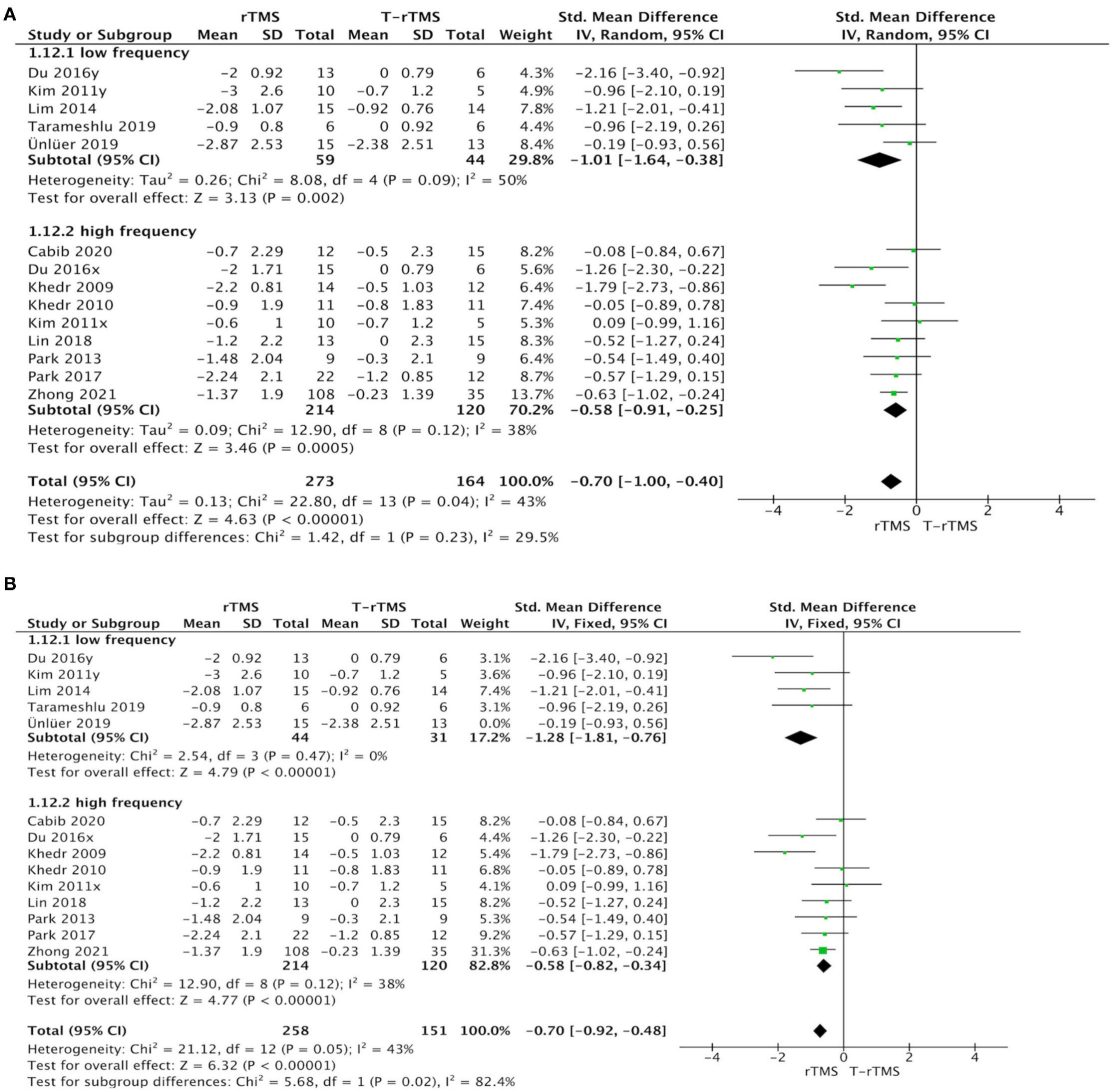
Subgroup analysis of primary outcomes. **(A)** Forest plot of the standardized mean difference (SMD) for change scores in dysphagia severity rating scales by the stimulation frequency. **(B)** Sensitive analysis (excluded the Ünlüer et al., 2019).

Stimulation frequency subgroup analysis showed that no significant difference was found between the low- (1 Hz) and high-frequency groups (≥3 Hz) (*p* = 0.23). Studies with low frequency (SMD = −1.01; 95% CI, −1.64 to −0.38, *p* = 0.002) and high frequency (SMD = −0.58; 95% CI, −0.91 to −0.25, *p* < 0.001) both indicated a higher effect size than the control conditions (Figure 5A).

#### Stimulation Location

Stimulation location subgroup analysis showed that both the affected hemisphere stimulation (SMD = −0.73; 95% CI, −1.21 to −0.26, *p* = 0.002) and the unaffected hemisphere stimulation (SMD = −1.07; 95% CI, −1.45 to −0.69, *p* < 0.001) had a higher effect size than the control conditions. However, no significant difference was found between the two groups (*p* = 0.28) (Table 2).

#### Stimulation Pattern

**Figure 6:**
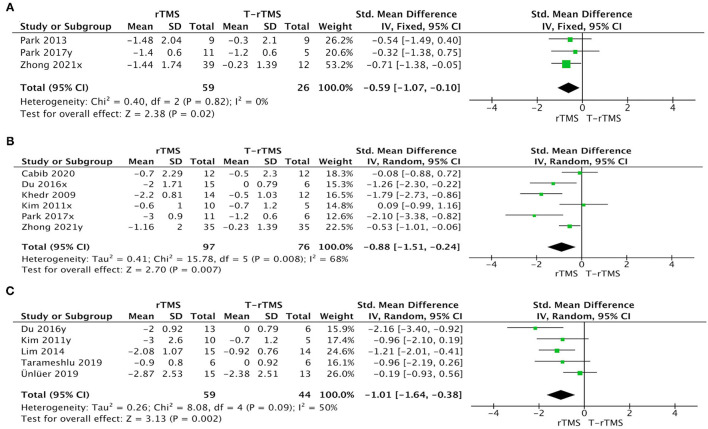
Subgroup analysis of primary outcomes: forest plot of the standardized mean difference (SMD) for change scores in dysphagia severity rating scales by the stimulation pattern. **(A)** High-frequency in unaffected hemisphere. **(B)** High-frequency in affected hemisphere. **(C)** Low-frequency in unaffected hemisphere.

The rTMS group exhibited a significant effect size than the control condition when patients adopted a high frequency in the affected hemisphere (SMD = −0.88; 95% CI, −1.51 to −0.24, *p* = 0.007), a high frequency in the unaffected hemisphere (SMD = −0.59; 95% CI, −1.07 to −0.10, *p* = 0.02), and a low frequency in the unaffected hemisphere (SMD = −1.01; 95% CI, −1.64 to −0.38, *p* = 0.002) (Figure 6).

#### Age

Subgroup analysis based on mean age also showed no significant difference (*p* = 0.08). Studies with a mean age of patients ≥65 years old (SMD = −0.41; 95% CI, −0.75 to −0.40, *p* = 0.02) and <65 years old (SMD = −0.93; 95% CI, −1.40 to −0.46, *p* = 0.001) both showed significant effect size compared with the control conditions (Table 2).

### Sensitivity Analyses

Sensitivity analyses for dysphagia rating scales change in PSD by excluding those studies with low quality (Khedr et al., 2009; Ünlüer et al., 2019). The effect size was much lower (SMD = −0.11; 95% CI, −0.57 to 0.34, *p* = 0.63), with low heterogeneity (*I*^2^ = 0%, *p* = 0.97) in the <5-day group (Figure 3B). Similarly, the effect size was much higher (SMD = −1.28; 95% CI, −1.81 to −0.76, *p* < 0.001) in the low-frequency group, with low heterogeneity (*I*^2^ = 0%, *p* = 0.47) (Figure 5B).

### Risk of Bias for Independent Studies

**Figure 7:**
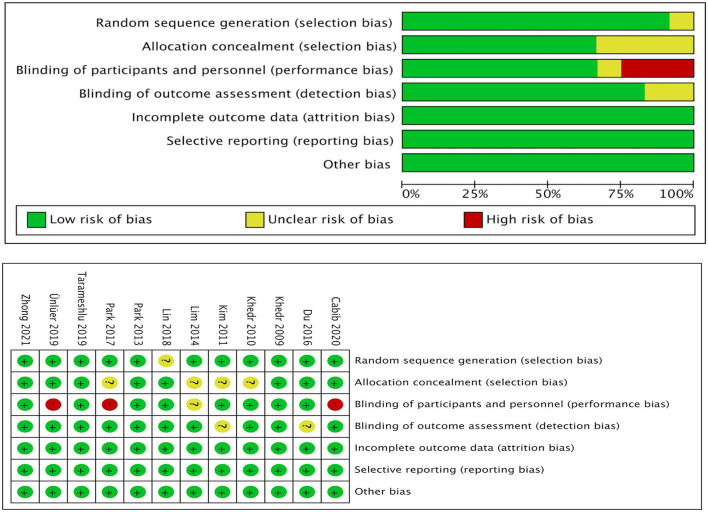
Risk of bias analysis and quality assessment of included trials. *Percentages of the assessments of each risk of bias item across all included studies.

**Figure 8:**
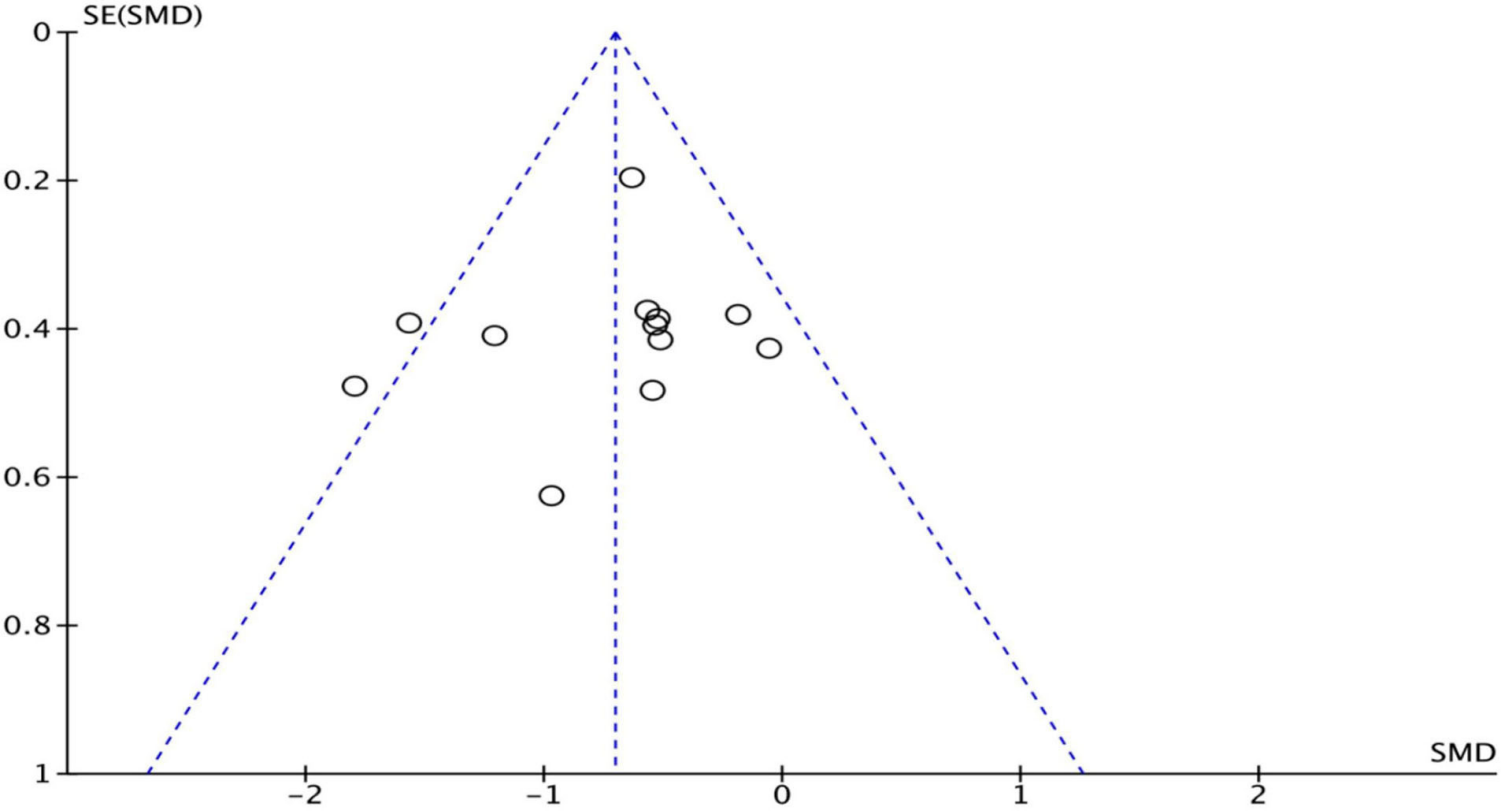
Funnel plot.

In the “risk of bias” graph, the overall quality of the literature was below the average (Figure 7). Eleven of the studies described the methods of random sequence generation (selection bias) in detail and were classified as low risk. The funnel plot indicated there may be publication bias (Figure 8), but the results must be viewed with caution due to the small number of publications.

## Discussion

The important role of rTMS for PSD treatment has been frequently confirmed by previous studies (Liao et al., 2016; Chiang et al., 2018). Even though some mild adverse events have been reported, such as mild or transient headache, tingling sensation, dizziness (Lim et al., 2014; Du et al., 2016; Ünlüer et al., 2019; Cabib et al., 2020; Zhong et al., 2021), etc., the effect of rTMS on PSD remains worthy of recognition. We conducted a meeting with different subgroups and discussed the optimal parameters of rTMS for PSD, which might have the potential to guide the clinical trial design and treatment strategies. The two significant findings of this meta-analysis were the positive therapeutic effect in an intervention lasting for >5 days and in the subacute phase after stroke.

Subgroup analysis for treatment duration suggested that the effect of >5 days was significantly superior to the control conditions, and there remained no effect when stimulation duration was <5 days. Therefore, the former treatment duration seems to be the more suitable rTMS treatment strategy for PSD. Gow et al. (2004) found that rTMS could regulate the excitability of the swallowing-related cerebral cortex with different frequency stimulations. The cortical excitability would disappear when the N-methyl-D-aspartate receptor was blocked, indicating that the influence of rTMS may be mediated by cortical synaptic function (Stefan et al., 2002). Hence, rTMS intervention for >5 days may have a cumulative effect on the cortical synaptic function and lead to the rehabilitation of PSD after appropriate intervention duration. However, it was difficult to identify whether the longer-term intervention was the moderator of better treatment effects or not because most intervention durations were too short to carry out reliable analysis among the included studies (the longest was only10 days) (Kim et al., 2011; Park, 2013; Park et al., 2017; Zhong et al., 2021). More studies are urgently needed to explore the most suitable treatment duration for rTMS on the treatment of PSD.

Subgroup analysis for stroke stages revealed a statistically significant difference at the subacute phase after stroke in the rTMS group, while no difference at the recovery phase, suggesting that the earlier rTMS intervention may be meaningful for the treatment of PSD. Hamdy et al. (1998) found that an increase in cortical excitability in the unaffected hemisphere was linked to the recovery of swallowing function. The early rTMS intervention for PSD might promote this compensatory mechanism, making PSD patients recover better in the subacute phase after stroke. At present, research on rTMS applied to the acute phase of stroke remains insufficient (Carey et al., 2017). Due to data limitations, we were unable to complete the detailed analysis of rTMS in the acute phase, subacute phase, and recovery phase after PSD.

Subgroup analysis for stimulation frequency indicated that both low- and high-frequency stimulations exhibited a significant effect size. Different frequencies of rTMS have been used to achieve the purpose of treatment, in which low-frequency rTMS mainly inhibit neural activity (Hummel et al., 2005; Mansur et al., 2005; Fregni et al., 2006), while high-frequency could improve motor excitability (Peinemann et al., 2004). Two key parameters of rTMS are the stimulation frequency and location; thus, the selection of stimulation site is usually highly related to the applied rTMS frequency. Some studies adopted low-frequency rTMS for the unaffected hemisphere to reduce the excitability of cortical neurons and tend to recover interhemispheric imbalance after a stroke, while the others chose to utilize high-frequency rTMS to stimulate the affected hemisphere (Kim et al., 2011; Du et al., 2016; Liao et al., 2016; Tarameshlu et al., 2019; Ünlüer et al., 2019). Interestingly, in this meta-analysis, we found that both low- and high-frequency stimulation could achieve a therapeutic effect, although the suitable frequency of rTMS stimulation remained in question.

Subgroup analysis for stimulation pattern showed that low- and high-frequency interventions for the unaffected hemisphere both achieved desired clinical efficacy. rTMS could effectively improve the functional rehabilitation of PSD patients through the stimulation to the cerebral pharyngeal motor cortex (Verin and Leroi, 2009; Momosaki et al., 2014; Du et al., 2016). The output signals of affected hemispheres reduced dramatically, and the balance between the bilateral hemispheres would be broken after stroke. The relatively excessive inhibition from the affected hemispheres might further disturb the balance of the brain (Alia et al., 2017). Promoting and optimizing rehabilitation strategies for PSD is closely associated with the revision of the balance of bilateral hemispheres, and the excitatory stimulation to unaffected hemispheres could accelerate the recovery process of PSD (Hamdy et al., 1998). We found that delivering the intervention to the unaffected hemisphere with high and low, or high frequency in the affected hemisphere all achieved expected efficacy. As the mechanism of rTMS has not yet been fully understood, further high-quality studies are needed to verify it.

However, we must acknowledge some certain limitations in this study. First, publication bias was quite obvious (Figures 7, 8), and the number of included articles was extremely limited. Second, the PSD outcome indicators were mainly DG and PAS scores, which might not be the most reasonable tools for the evaluation of swallowing function. Finally, the severity of primary diseases was not distinguished, which may affect the effect size, reliability, validity, and quality of this meta-analysis. Therefore, the interpretation of the results should be made more cautious, and more high-quality RCT literature analyses are urgently needed to confirm the conclusions.

## Conclusion

rTMS could bring new strategies and rational therapeutics to the management of PSD patients. Early intervention of rTMS after stroke and the treatment duration of more than 5 days may provide a meaningful effect for PSD patients returning to normal life and work activities. However, there are still controversies about the best stimulation hemisphere and frequency of rTMS at present.

## Data Availability Statement

The original contributions presented in the study are included in the article/Supplementary Material, further inquiries can be directed to the corresponding author.

## Author Contributions

JQ responsible for article retrieval and writing. Z-mW, Q-pY, and YD responsible for the selection of articles and analysis the data. Z-lD responsible for the review of articles and ensuring that all listed authors have approved the manuscript before submission. All authors contributed to the article and approved the submitted version.

## Funding

This work has been sponsored by the Natural Science Foundation of China (NSFC, No. 81972159) and Natural Science Foundation of Guangdong Province (NSFP, No. 2020A1515010881).

## Conflict of Interest

The authors declare that the research was conducted in the absence of any commercial or financial relationships that could be construed as a potential conflict of interest.

## Publisher's Note

All claims expressed in this article are solely those of the authors and do not necessarily represent those of their affiliated organizations, or those of the publisher, the editors and the reviewers. Any product that may be evaluated in this article, or claim that may be made by its manufacturer, is not guaranteed or endorsed by the publisher.

## Abbreviations

PSD: poststroke dysphagia
rTMS: repetitive transcranial magnetic stimulation
DG: dysphagia grade
PAS: Penetration Aspiration Scale
T-rTMS: none repetitive transcranial magnetic stimulation
VFSS: Video Fluoroscopic Swallowing Study
RCT: randomized controlled trials
FEES: fiberoptic endoscopic evaluation of swallowing
NIBS: noninvasive brain stimulation
tDCS: transcranial direct current stimulation.
