# Supplementary material for: The Effect and Optimal Parameters of Repetitive Transcranial Magnetic Stimulation on Poststroke Dysphagia: A Meta-Analysis of Randomized Controlled Trials
Source: Front Neurosci. 2022 Apr 28;16:845737. doi: 10.3389/fnins.2022.845737 (PMC9095943; doi:10.3389/fnins.2022.845737)
Supplement: Supplementary file 1 [file Data_Sheet_1.docx]

**Supplemental Appendix**

**Study identification and search method**

**Pubmed:**

(*dysphag* OR *swallow* OR deglutition OR *pharyng* OR esophageal ORoropharyngeal) AND ( transcranial magnetic stimulation[MESH] OR *TMS) AND (placebo OR controlled OR double-blind OR single-blind OR clinical trial OR random* OR RCT*)

**Embase:**

#1 (‘dysphagia’/exp OR dysphagia) AND (‘transcranial magnetic stimulation’/exp OR transcranial magnetic stimulation OR *TMS) AND (placebo OR controlled OR double-blind OR single-blind OR clinical trial OR random* OR RCT*)

**Cochrane:**

#1 *dysphag* OR *swallow* OR deglutition OR *pharyng* OR esophageal ORoropharyngeal

#2 transcranial magnetic stimulation OR *TMS

#3 placebo OR controlled OR double-blind OR single-blind OR clinical trial OR random* OR RCT*

#4 MeSH descriptor: [transcranial magnetic stimulation] explode all trees

#4 #1 AND (#2 OR #3) AND #4

**Web Of Science:**

TS= (dysphagia OR swallowing disorders OR deglutition disorders OR swallowing dysfunction

OR deglutition OR esophageal OR oropharyngeal) AND (TMS OR rTMS OR magnetic OR transcranial OR transcranial magnetic stimulation) AND (placebo OR controlled OR double-blind OR single-blind OR clinical trial OR random OR randomly OR randomization OR RCT OR RCTs)
